# Experts’ recommendations for the management of cardiogenic shock in children

**DOI:** 10.1186/s13613-016-0111-2

**Published:** 2016-02-16

**Authors:** Olivier Brissaud, Astrid Botte, Gilles Cambonie, Stéphane Dauger, Laure de Saint Blanquat, Philippe Durand, Véronique Gournay, Elodie Guillet, Daniela Laux, Francis Leclerc, Philippe Mauriat, Thierry Boulain, Khaldoun Kuteifan

**Affiliations:** Unité de Réanimation Pédiatrique et Néonatale, Hôpital des Enfants, CHU Pellegrin Enfants, Place Amélie Raba Léon, 33000 Bordeaux, France; Unité de Réanimation Pédiatrique, Faculté de Médecine, Université de Lille Nord de France, Hôpital Jeanne de Flandre CHU de Lille, 54, Avenue Eugène Avinée, 59037 Lille Cedex, France; Département de Pédiatrie Néonatale et Réanimations, Pôle Hospitalo-Universitaire Femme-Mère-Enfant, Hôpital Arnaud-de-Villeneuve, Centre Hospitalier Régional Universitaire de Montpellier, 371, Avenue du Doyen-Gaston-Giraud, 34295 Montpellier Cedex 5, France; Réanimation et Surveillance Continue Pédiatriques, Pôle de Pédiatrie Médicale, Hôpital Robert-Debré, Assistance Publique-Hôpitaux de Paris, Université Paris Diderot-Paris 7, Sorbonne Paris Cité, 48, Boulevard Sérurier, 75019 Paris, France; Service de Réanimation, CHU Necker-Enfants-Malades, 149, rue de Sèvres, 75743 Paris Cedex 15, France; Réanimation Pédiatrique, AP-HP, CHU Kremlin Bicêtre, 78, rue du Général Leclerc, 94270 Le Kremlin Bicêtre, France; Service de Cardiologie Pédiatrique, CHU de Nantes, 44093 Nantes Cedex, France; Pôle des Cardiopathies Congénitales, Centre Chirurgical Marie Lannelongue, 133, Avenue de la Résistance, 92350 Le Plessis-Robinson, France; Service de Cardiologie Pédiatrique et Congénitale, Hôpital Haut-Lévèque, CHU de Bordeaux, Avenue de Magellan, 33604 Pessac Cedex, France; Service de Réanimation Polyvalente, Hôpital de La Source, Centre Hospitalier Régional Orléans, 45067 Orléans, France; Service de Réanimation Médicale, Hôpital Émile-Muller, 68070 Mulhouse, France

## Abstract

Cardiogenic shock which corresponds to an acute state of circulatory failure due to impairment of myocardial contractility is a very rare disease in children, even more than in adults. To date, no international recommendations regarding its management in critically ill children are available. An experts’ recommendations in adult population have recently been made (Levy et al. Ann Intensive Care 5(1):52, 2015; Levy et al. Ann Intensive Care 5(1):26, 2015). We present herein recommendations for the management of cardiogenic shock in children, developed with the grading of recommendations’ assessment, development, and evaluation system by an expert group of the Groupe Francophone de Réanimation et Urgences Pédiatriques (French Group for Pediatric Intensive Care and Emergencies). The recommendations cover four major fields of application such as: recognition of early signs of shock and the patient pathway, management principles and therapeutic goals, monitoring hemodynamic and biological variables, and circulatory support (indications, techniques, organization, and transfer criteria). Major principle care for children with cardiogenic shock is primarily based on clinical and echocardiographic assessment. There are few drugs reported as effective in childhood in the medical literature. The use of circulatory support should be facilitated in terms of organization and reflected in the centers that support these children. Children with cardiogenic shock are vulnerable and should be followed regularly by intensivist cardiologists and pediatricians. The experts emphasize the multidisciplinary nature of management of children with cardiogenic shock and the importance of effective communication between emergency medical assistance teams (SAMU), mobile pediatric emergency units (SMUR), pediatric emergency departments, pediatric cardiology and cardiac surgery departments, and pediatric intensive care units.

**Electronic supplementary material:**

The online version of this article (doi:10.1186/s13613-016-0111-2) contains supplementary material, which is available to authorized users.

## Background

Cardiogenic shock is an acute state of circulatory failure due to impairment of myocardial contractility. In children, the clinical signs of cardiac failure are tachycardia, dyspnea, and hepatomegaly, together with global signs related to a decrease of cardiac output. Cardiogenic shock represents 5–13 % of diagnosed cases of shock in pediatric emergencies [3, 4]. It is the most advanced and most serious stage of heart failure. In hospitalized children, cardiogenic shock is lethal in 5–10 % of cases, a mortality rate similar to that observed in adults [5, 6]. Extracardiac comorbidities (such as sepsis, acute kidney failure, and liver failure) can lead to a fivefold increase of the mortality rate [5, 6]. The following causes of heart failure are the most likely to lead to cardiogenic shock [5–10]: primary or secondary cardiomyopathy, acute or fulminant myocarditis, arrhythmia, congenital heart disease (whether surgically managed or not), postoperative period after cardiac surgery, and, in exceptional cases, endocarditis, rheumatic fever, severe Kawasaki disease, stress cardiomyopathy (Tako-Tsubo), valve cord rupture, drug or toxic substance intoxication. Cardiogenic shock can also be caused by extracardiac diseases (sepsis-induced myocardial failure, pulmonary embolization, pneumothorax, tamponade). Mortality is directly related to the underlying disease: congenital heart disease, rhythm disorders, acquired heart diseases, and cardiomyopathies, in 4.7, 23, 8.7, and 25 % of children, respectively [8, 11]. Mortality in case of decompensation is similar in children and adults. This is related to the fact that children also have other comorbidities but different from those of adults.

Management and monitoring of cardiogenic shock in childhood differ between anatomically normal heart and congenital heart disease. Recommendations made in this document are addressed to children without congenital heart disease; it is important in the case of children with heart disease who decompensates to be in contact with pediatric cardiologists to optimize management. Cardiogenic shock occurs when heart and circulation are no longer able to adapt to the situation and is characterized by severely impaired myocardial contractility, increased preload, severely impaired myocardial compliance, increased afterload, and an abnormally and persistently high heart rate. Decompensation may occur and manifests as a drop in blood pressure and/or cardiac output with inadequate tissue perfusion, as this is the final goal of cardiovascular performance, and the onset of anaerobic metabolism with the production of lactic acid. Unless treated, cardiogenic shock leads to multiple organ failure then progresses rapidly to death.

Even if the literature is poor regarding strong evidences in management of cardiogenic shock, we report, in this article, recommendations built by pediatricians and anesthesiologists, experts in the topic of cardiogenic shock. Process of recommendations’ elaboration permitted us to avoid making the recommendations only based on own experience. The recommendations cover four major fields of application such as: recognition of the early signs of shock and the patient pathway, management principles and therapeutic goals, monitoring hemodynamic and biological variables, and circulatory support (indications, techniques, organization, and transfer criteria).

## Field of application 1: recognizing the early signs of shock

**Fig. 1:**
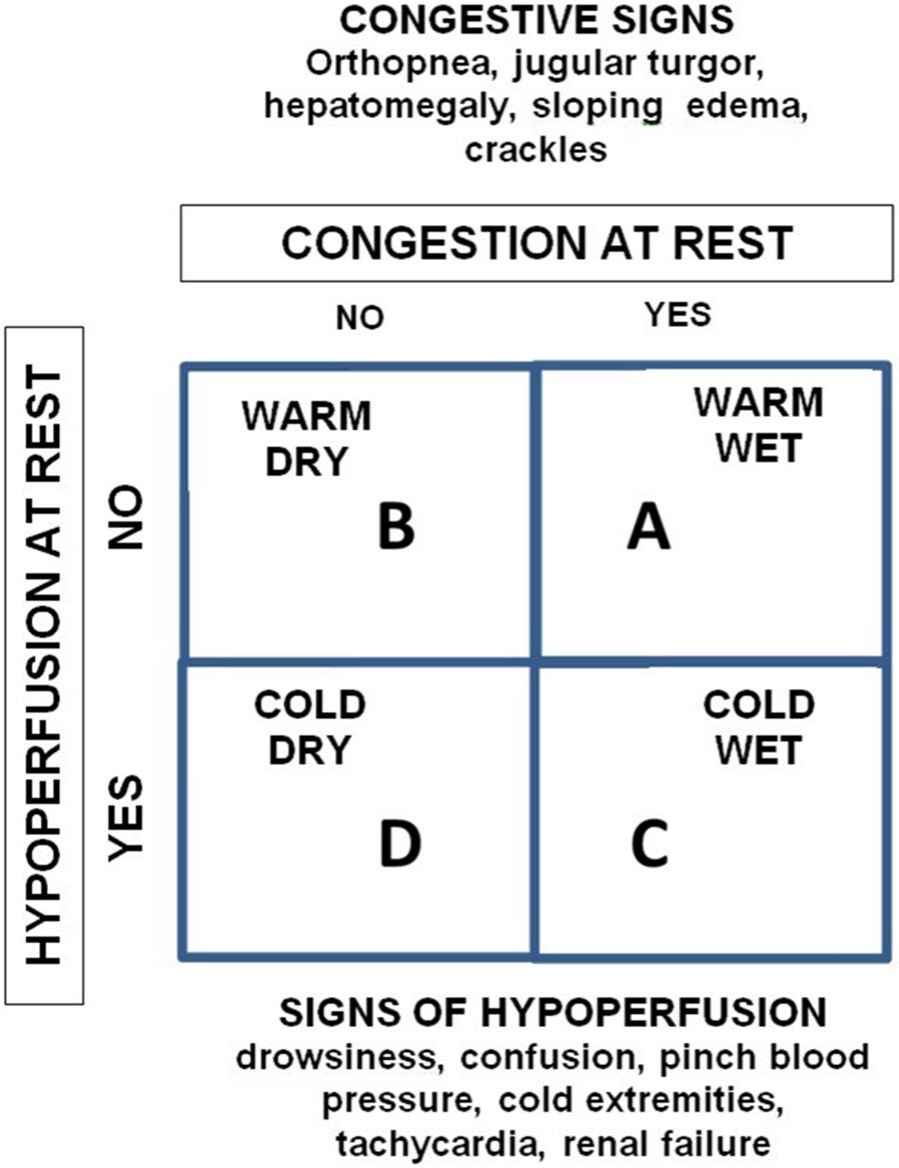
Cardiogenic shock—Warner Stevenson concept (2)

Three pathophysiological states have been described for cardiogenic shock (Fig. 1):

*Cold and wet*: reduced myocardial contractility and increased left ventricular filling pressure (systolic and diastolic dysfunction).

*Cold and dry*: reduced myocardial contractility and low left ventricular filling pressure (systolic dysfunction).

*Warm and wet*: normal myocardial contractility and high left ventricular filling pressure (diastolic dysfunction).

### Clinical signs (Table 1)

Impaired consciousness, arterial hypotension, and respiratory distress with a fall in oxygen saturation are the early signs of decompensated heart failure and cardiogenic shock [12–14].

**Table 1:** Nonspecific clinical signs of cardiogenic shock in children

General	Default weight gain, asthenia Whiteness, sweating (diaphoresis) marbling ends
Hemodynamics	Tachycardia, edema Decreased peripheral pulses Heart murmur, gallop rhythm Bradycardia, arrhythmia Jugular turgor
Respiratory	Polypnea or bradypnea; crackles Cyanosis of the extremities or peri-oral
Neurological	Anxiety, restlessness Confusion Drowsiness Convulsions Coma
Digestive	Hepatomegaly Decreased transit Food difficulties (polypnea, sweating during feeding)
Kidney	Oliguria Anuria

### Etiological signs

Such signs are based on the patient’s medical/surgical history (heart disease, systemic disease, long-term heart failure), but also on blood pressure measurements in all limbs, as well as the presence of heart murmur, arrhythmia, or sepsis.

### Para-clinical signs

Chest X-rays (cardiomegaly, pulmonary edema), ECGs (arrhythmia, myocardial ischemia, conduction disorders, drugs effect, changes in the QT interval), and transthoracic echocardiograms are useful for diagnostic, etiological, therapeutic, and prognostic purposes.

### Complications

In addition to the risk of multiple organ failure and death, cardiogenic shock is sometimes associated with cardiorenal syndrome (oliguria, increased urea and creatinine levels concomitant with myocardial failure), which leads to longer hospital stays, the need for circulatory support, and an increased death rate [15].

### First-line treatment [12, 13, 16–18]

The aim of initial patient management is to restore adequate oxygen delivery to peripheral tissues. This relies on emergency support (early recognition, monitoring, access), optimizing ventilation/gas exchange (oxygen therapy ± noninvasive or invasive ventilation with a saturation objective of >95 % except in cyanotic heart disease patients), optimizing the preload and afterload (volume expansion or diuretics and fluid restriction, inotropes, discontinuation of deleterious medication), and treating curable causes (fluid and electrolyte balance, rhythm, or thromboembolic disorders; pneumothorax, tamponade, infection).

## Field of application 2: patient pathway, management principles, and therapeutic goals

### Patient pathway

- All patients with cardiogenic shock should be transferred to PICU (*strong agreement*).
- Children with cardiogenic shock should be managed by a chain of specialized medical teams which adapt their care to the real or potential gravity of the patient throughout his/her stay in care. This medical chain should be clearly identified by all healthcare actors (SAMU, SMUR, pediatric emergency departments, pediatric cardiology and cardiac surgery departments, and PICU) (*strong agreement*).
- A telephone hotline to an “expert center” providing specialized responses should be available 24/24 (*strong agreement*).
- It is strongly advised that regional health agencies officially designate “expert centers” (*strong agreement*).
- The following multidisciplinary expertise should be available in such expert centers: pediatric medical and interventional cardiology, pediatric anesthesia, pediatric thoracic, vascular and cardiac surgery, pediatric intensive care, radiology (notably for interventional vascular procedures), mobile circulatory support unit (*strong agreement*).

### Does prehospital care such as European pediatric life support improve the prognosis of cardiogenic shock?

- Basic or advanced European pediatric life support resuscitation, either prior to arrival or at the hospital should be used to reduce morbidity and mortality of cardiogenic shock (*strong agreement*).
- It is probably not advisable to use high doses of adrenaline (≥30 µg/kg) during resuscitation compared with usual doses (*strong agreement*).
- Early goal-directed therapy should be based on clinical (diuresis, preload, blood pressure) and laboratory (blood pH, lactate levels, continuous venous oxygen saturation) variables as well as echocardiogram (*strong agreement*).
- Once the acute phase of cardiogenic shock has been relieved, the patient should be prescribed appropriate oral therapy to treat cardiac insufficiency and should be closely monitored (*strong agreement*).

For non-trauma-related cardiogenic shock, “advanced pediatric life support” is associated with reduced mortality [OR 0.58 (0.4–0.84)] and functional morbidity [OR 0.13 (0.04–0.41)] [19]. No benefit has been observed when high doses of adrenaline (0.1 mg/kg IV) are injected compared with usual doses (0.01 mg/kg IV) during prehospital or hospital management of cardiac arrest [20, 21]. Implementing therapeutic goals during shock management improves patient survival, notably for septic shock [22, 23].

### Medications

#### Usefulness of fluid resuscitation in cardiogenic shock

- Fluid resuscitation should only be indicated in patients with cardiogenic shock after clinical assessment (preload insufficiency); it is advised that echocardiographic evaluation be used during such assessment (*strong agreement*).
- Fluid resuscitation with colloids and/or crystalloids should not be used in patients with cardiogenic shock (*strong agreement*).

In the FEAST study, despite specific comorbid conditions compared to those seen in industrialized countries, the increased mortality of children receiving fluid boluses was found to be due to cardiac events (cardiogenic shock) related to fluid overload [24].

#### Usefulness of diuretics in the management of cardiogenic shock

- In children with fluid overload and ventricular dysfunction, diuretics (such as furosemide) should be used to return to euvolemic state while monitoring clinical criteria and measuring the cardiac output (low level of evidence, *strong agreement*).
- It is probably not advisable to use venous perfusions of nesiritide (recombinant BNP) in children with cardiogenic shock (*strong agreement*).
- It is probably not advisable to use tolvaptan, a selective antagonist of the arginine vasopressin receptors (V2) that is given orally in children with cardiogenic shock (*strong agreement*).

In children with heart failure already treated with one or more inotropes and a loop diuretic, continuous perfusion of 0.005–0.02 µg/kg/min nesiritide improves the fluid balance and reduces pressure in the right atrium after 24 h [25]. Other studies have shown that the continuous perfusion of 0.01–0.03 µg/kg/min nesiritide improves echocardiogram variables (heart rate, left ventricular ejection fraction) and laboratory values (decreased creatinine, BNP and aldosterone levels) after 72 h [26]. In a retrospective series of 28 children with heart failure and hyponatremia, tolvaptan, a selective non-peptide antagonist the arginine vasopressin receptors (V2), improved, when used orally at a dose of 0.3 mg/kg, both diuresis and sodium levels after 72 h [27].

#### Usefulness of inotropes in the management of cardiogenic shock

- Dobutamine should be used at a dose of 5–20 µg/kg/min in cases of cardiogenic shock with maintained blood pressure so as to restore cardiac output (*strong agreement*).
- It is probably advisable to use milrinone (continuous perfusion of 0.5–0.75 µg/kg/min) as an alternative to dobutamine in children with cardiogenic shock, especially in post-cardiac surgery patients and in cases with impaired right ventricle function and/or associated pulmonary arterial hypertension (*strong agreement*).
- Levosimendan (continuous perfusion of 0.1–0.2 µg/kg/min) may be used as second-line treatment in patients with cardiogenic shock who do not respond to the usual therapy (dobutamine or milrinone with or without diuretics), especially for postoperative management after cardiac surgery (*strong agreement*).

Dobutamine is still the gold standard inotrope for the dynamic assessment of the cardiovascular system and is used as the reference inotrope in comparative studies [28]. After cardiac surgery, low doses (2.5 µg/kg/min) of dobutamine increase the cardiac index and high doses (20 µg/kg/min) improve the cardiac index in patients with Alagille syndrome [29, 30]. Its hemodynamic effects are identical to those of dopamine but without leading to increased pulmonary vascular resistance [31]. A review has been recently published regarding pharmacokinetics and pharmacodynamics of dobutamine in neonates, including studies in children [32].

When used to treat septic shock with normo/hypokinesia and compared with a placebo, milrinone (loading dose of 50 µg/kg, followed by continuous perfusion of 0.5 µg/kg/min for 2 h) increases the cardiac index, stroke volume index, and oxygen delivery (DO_2_) and reduces systemic and pulmonary vascular resistance and the mean pulmonary arterial pressure 30 min, 1 h, and 2 h after a loading dose of 50 µg/kg [33]. Bailey et al. [34] demonstrated that the standardized loading dose of 50 µg/kg over 5 min was associated with a 12 % decrease of the mean arterial pressure and an 18 % increase of the cardiac index. Some authors have compared a placebo group with both a “low dose” group (25 µg/kg over 60 min, followed by continuous perfusion of 0.25 µg/kg/min for 35 h) and a “high dose” group (75 µg/kg over 60 min, followed by continuous perfusion of 0.75 µg/kg/min for 35 h). The high dose of milrinone reduced the risk of low cardiac output by 55 % at 36 h (*p* = 0.023) and by 48 % at 30 days (*p* = 0.049) [35]. In practice, the loading dose of milrinone has been abandoned due to the risk of vasoplegia. In a series of 40 post-cardiac surgery children, Lechner et al. [36] found that levosimendan (0.1 µg/kg/min) had a greater effect than milrinone (0.5 µg/kg/min) on cardiac index and cardiac output. In 15 children with acute heart failure, levosimendan was associated with an improved left ventricular end systolic fraction and allowed the patients to be given lower doses of catecholamines [37]. As for milrinone, in practice, the loading dose of levosimendan has been abandoned due to the risk of vasoplegia.

#### Usefulness of vasopressors in the management of cardiogenic shock

- If vasopressors are combined with inotropes to achieve adequate perfusion pressure, it is probably advisable to use noradrenaline as first-line treatment, especially in patients with low cardiac output, reduced vascular resistance, and persistent hypotension (*strong agreement*).
- Noradrenaline should be replaced by adrenaline in patients with inotrope-resistant cardiogenic shock (*strong agreement*).
- It is probably not advisable to use dopamine to treat cardiogenic shock in children (*strong agreement*).
- It is probably advisable to use terlipressin/arginine vasopressin as last-resort treatment in children with vasopressor-resistant cardiogenic shock (common vasopressors such as noradrenaline and adrenaline) (*strong agreement*).

In 2011, Levy et al. [38] demonstrated the advantage of combining the use of norepinephrine, dobutamine, and dopamine in adult patients with cardiogenic shock. Two forms of the arginine vasopressin (AVP) hormone are used in clinical practice: exogenous AVP and terlipressin, a synthetic analog with a longer half-life (6 h vs. 24 min for AVP). There are thirty-five reports in the literature on its clinical use in neonates, children, and adolescents; seven of the studies investigated its use in patients with low cardiac output and/or severe vasodilation after cardiac surgery, and four for cardiopulmonary resuscitation [39]. In the context of pediatric cardiac surgery (neonates and children), it seems more likely that the positive effects of exogenous AVP [40] and terlipressin [41] are due to restored vascular tone than to improved left ventricular function [42, 43]. However, analysis of the pediatric cases recorded in the North American registry of cardiopulmonary resuscitated patients suggested that AVP had a deleterious effect on the possibility of return of spontaneous circulation [44].

#### Usefulness of vasodilators in the management of cardiogenic shock

- It is probably not advisable to use nitrated derivatives to treat cardiogenic shock in children (*strong agreement*).

Several series of cases evidenced a positive effect when nitroprussiate was used to treat patients with low cardiac output after cardiac surgery [45, 46]. Similar findings were reported for acute cardiac failure [47].

#### Usefulness of antiarrhythmic agents in the management of cardiogenic shock

- Beta-blockers should not be used to treat cardiogenic shock in children (*strong agreement*).

Meta-analysis of three studies assessing the effect of beta-blockers in children with chronic congestive heart failure produced conflicting results. The study with the largest population (161 children) demonstrated the non-superiority of carvedilol over placebo using a composite score for heart failure [48]. Another study suggested the superiority of procainamide over amiodarone for emergency treatment of recurring supraventricular tachycardia [49].

### Other therapeutic principles

#### Usefulness of sedation/analgesia in cardiogenic shock

- No expert recommendations

#### Usefulness of anticoagulants/anti-aggregation agents in cardiogenic shock

- No expert recommendations

#### Usefulness of therapeutic hypothermia in cardiogenic shock

- No expert recommendations

#### Usefulness of continuous renal replacement therapy in cardiogenic shock

- No expert recommendations

#### Usefulness of transfusion in cardiogenic shock

- No expert recommendations

#### Usefulness of immunoglobulins in the management of cardiogenic shock in children with myocarditis

- In cases of acute myocarditis with cardiogenic shock, it is probably not advisable to use immunoglobulins (*strong agreement*).

According to a recent report, fulminant myocarditis results in cardiogenic shock in 28 % of cases [9]. No prospective randomized pediatric studies have assessed the use of immunoglobulins to treat acute myocarditis [50]. The largest multicenter trial, a retrospective study on 100 patients, showed that treatment with immunoglobulins did not improve pediatric patient survival, whatever the severity score [51].

#### Usefulness of immunosuppressive therapy in the management of cardiogenic shock in children with myocarditis

- In cases of acute myocarditis with cardiogenic shock, it is probably not advisable to use immunosuppressant drugs (*strong agreement*).

Meta-analysis of 9 studies (a total of 139 children treated with different immunosuppressant drugs) showed no benefit of immunosuppressive therapy in patients with myocarditis [52]. Two randomized prospective studies showed a favorable effect on restoring left ventricular function in children with chronic heart failure following myocarditis [53, 54], but combined the use of variable doses of corticoids, azathioprine, and ciclosporine.

#### Comparison of the usefulness of noninvasive ventilation versus invasive ventilation in the management of cardiogenic shock

- Noninvasive ventilation should not be preferred over invasive ventilation in children with cardiogenic shock (*strong agreement*).

No studies have compared the use of noninvasive ventilation and invasive ventilation in children with acute heart failure. Noninvasive ventilation (continuous positive airway pressure or Bi-level airway pressure) is effective in 66–80 % of cases when used in pediatric post-cardiac surgery patients, notably in the presence of atelectasis or pulmonary edema [55, 56]. A team from Turkey reported the use of a noninvasive ventilation with a helmet in 3 children aged 18 months, 5 years, and 7 years with cardiogenic pulmonary edema following scorpion envenomation [57].

#### Usefulness of cardiac resynchronization therapy in the management of cardiogenic shock

- Cardiac resynchronization therapy should not be used in children with cardiogenic shock (low overall level of evidence, *strong agreement*).

No studies have investigated the usefulness of cardiac resynchronization therapy in pediatric patients with cardiogenic shock. Nevertheless, few studies have been carried out on children with severe acute heart failure following cardiac surgery. These studies are based on small numbers of hemodynamically stable patients and did not include control groups [58–62].

## Field of application 3: monitoring hemodynamic and biological variables

### Usefulness of clinical monitoring in the management of cardiogenic shock

- Patients with cardiogenic shock should be clinically examined several times a day to evaluate the effectiveness or in-effectiveness of treatment (pulse volume, peripheral perfusion, heart rate, preload, hepatomegaly, auscultation crackles, distended jugular veins, edema syndrome) (*strong agreement*).
- Monitoring data for children with cardiogenic shock should also include hourly diuresis measurements and the input/output balance (*strong agreement*).

On auscultation of children with cardiogenic shock, tachycardia, gallop rhythm, heart murmur, and signs of lung congestion can be detected. Peripheral pulses are often weakest. Hepatomegaly, distended jugular veins, and peripheral edema are nearly always detected in children with cardiogenic shock [63]. In infants, supraventricular tachycardia should be suspected if tachycardia with a heart rate >220 bpm occurs suddenly.

### Usefulness of chest X-rays in the management of cardiogenic shock

- The initial examination of cardiogenic shock patients should include a chest X-ray (low level of evidence, *strong agreement*).

Chest X-ray generally shows cardiomegaly (antero-posterior projection, end-inspiration; cardiothoracic ratio >0.6 in neonates, >0.55 in infants, and >0.5 in children). Chest X-ray is used to assess the state of the parenchyma and lung vessels. They can evidence perihilar fluffy opacities with butterfly/bat wing patterns (pulmonary edema of cardiogenic origin), the water bottle sign (pericardial effusion), or a boot-shaped heart (right ventricular dilatation) [63–66].

### Usefulness of monitoring laboratory values in the management of cardiogenic shock

- Arterial pH and blood lactate levels should be determined repeated to assess the course of shock and evaluate the efficacy of therapeutic measures (*strong agreement*).
- The levels of the following variables should be quantified at regular intervals to assess organ dysfunction in patients with cardiogenic shock: plasma ions, blood urea and creatinine, blood glucose, transaminases (*strong agreement*).
- Repeated determination of CPK-MB levels
  - It is probably not advisable to determine blood CPK-MB levels in patients with cardiogenic shock (*strong agreement*).
- Repeated determination of NT-proBNP levels
  - BNP/NT-proBNP levels should be quantified at regular intervals in patients with cardiogenic shock to assess the severity of heart disease (*strong agreement*).
- Repeated determination of troponin levels
  - Troponin levels should be quantified at regular intervals in patients with cardiogenic shock to assess the severity of myocardial involvement as well as the response to treatment (*strong agreement*).

Blood lactate level, used as a predictor of mortality, is one of the therapy goals for sepsis management in both children and adults [67]; depending on the studies, the goal value ranges from 2 to 3 mmol/L [68, 69]. Increased lactate levels can be due to the use of inotropes (adrenaline, dobutamine), hyperventilation, and impaired mitochondrial function without circulatory collapse, and certain volume expansion fluids [70]. In patients with cardiogenic shock, the increase in CPK-MB is not correlated with the severity of myocardial involvement [71]. The different troponin isoforms are more specific and sensitive markers of acute myocardial involvement than CPK-MB [71]; even though to date and unlike what is observed in adults, the prognostic value of troponin is not clearly established in pediatric patients with cardiogenic shock [72]. In neonates and children, the normal values of troponin and CPK-MB are 0.2–0.4 and 0.6 µg/L, respectively. The prognostic value of BNP in children with cardiogenic shock has not been demonstrated formally even though its determination is still used widely to assess the severity of cardiac involvement, particularly in patients with preexisting cardiomyopathy [73]. A recent report based on 181 pediatric cases demonstrated the relevance of BNP when assessing the severity of congestive heart failure [74]. Gessler et al. [75] demonstrated that the increase in preoperative NT-proBNP levels was associated with the quantity of inotropes used during the postoperative period in 40 children who underwent cardiac surgery. However, these results have yet to be confirmed by other teams [76]. At the beginning of the twenty-first century, the use of NT-proBNP levels as a marker discriminating between the pulmonary and cardiac causes of dyspnea was assessed in children [77]. In adults with cardiogenic shock, BNP levels appear to correlate well with mortality and morbidity rates [78].

## Usefulness of hemodynamic monitoring in cardiogenic shock

### Usefulness of noninvasive hemodynamic monitoring in cardiogenic shock

#### Oxygen saturation: SpO2

- SpO2 values should be monitored continuously in children with cardiogenic shock (*strong agreement*).

SpO2 is an essential indicator for monitoring a patient with decompensated circulatory failure. Its value should be evaluated together with clinical assessment and arterial blood gas values (SaO2) [11, 79].

#### Electrocardiogram: ECG

- Patients with cardiogenic shock should undergo ECG examination in order to guide diagnosis (pericarditis, myocarditis, coronary ischemia, pulmonary embolization) and/or diagnose possible rhythm disorders that are causing cardiogenic shock (*strong agreement*).
- If in doubt, a pediatric cardiology team should be contacted to analyze the ECG (*strong agreement*).

An ECG is essential and should be repeated at regular intervals during the course of cardiogenic shock in children [63]. It may show abnormalities related to underlying heart disease [12], rhythm disorders due to cardiomyopathy [80, 81], supraventricular tachycardia or specific signs of an acute condition such as pericarditis (microvoltage), viral myocarditis (repolarization issues, ST-segment elevation or depression, related ventricular rhythm disorders), myocardial infarction in Kawasaki disease (Q wave, ST-segment depression), or drug intoxication [65].

#### Noninvasive arterial pressure

- We strongly recommend use of an arterial catheter to measure the arterial blood pressure in patients with decompensated circulatory failure as seen in cardiogenic shock (*strong agreement*).

Only invasive techniques provide reliable measurements in such patients with circulatory failure, especially when decompensated [82]. Noninvasive arterial blood pressure measurements can be complicated to obtain in neonates [83] and require special equipment. The authors of one study [84] advise using an arm cuff of width equal to 40 % of the upper arm circumference. However, this technique overestimates the diastolic blood pressure. If blood pressure is difficult to measure noninvasively in a child with impaired consciousness and an irregular O_2_ saturation curve, then hemodynamic failure can be suspected.

#### Usefulness of TTE monitoring in cardiogenic shock

- We strongly recommend that all PICU have a 24/7 transthoracic echocardiogram (TTE) service (*strong agreement*).
- In patients with acute circulatory failure as seen in cardiogenic shock, TTE should be used to assess cardiac function (*strong agreement*).
- We strongly advise contacting a pediatric cardiology team (by phone, bedside visit, etc.) if a child with cardiogenic shock is admitted to PICU (*strong agreement*).
- In cardiogenic shock patients, at least the following items should be assessed using TTE: systolic and diastolic function of both ventricles, pulmonary pressures and coronary visualization, confirmation of normal cardiac structure (*strong agreement*).
- We strongly advise use of TTE when performing pericardial puncture in patients with tamponade (ultrasound-guided drainage) (*strong agreement*).
- TTE should be performed if a patient suffers from cardiogenic shock following cardiac surgery (*strong agreement*).
- We strongly recommend that specific certification courses in TTE be implemented at the national level for neonatal and pediatric intensive care practitioners (*strong agreement*).

TTE is recommended in the management of septic shock [85]. Few studies focus on the diagnostic and prognostic value of TTE in pediatric cases of cardiogenic shock [86]. It is the ideal point-of-care technique [87]. TTE enables even those with minimal training to rapidly detect pericardial effusion or impaired left ventricular function [88, 89]. It can also be used to assess the efficacy of therapeutic measures and any related complications. In scorpion envenomation patients with authentic cardiogenic shock, it was shown that early observation of proper left ventricular function using TTE had a very good positive predictive value for recovery without heart failure [86]. TTE should be used at the earliest stage when possible in order to detect subclinical abnormalities. To monitor the cardiotoxicity of cytotoxic drugs, some authors recommend routine TTE assessment before administering the drugs and then at 1 week, 6 months, and 1 year [90]. It is essential that residents and senior residents receive training on functional TTE in intensive care [91].

#### Usefulness of near-infrared spectroscopy (NIRS) monitoring in cardiogenic shock

- Cerebral, mesenteric, or renal NIRS can be used to monitor organ perfusion in patients with cardiogenic shock (*strong agreement*).

NIRS measures oxygenation at the microcirculatory level in vessels of diameter <1 mm [92]. Its value varies from organ to organ and depends on each organ’s metabolism. This technique has mostly been used in unstable children undergoing cardiac surgery (intra- and postoperatively). During extracorporeal circulation, at the time of the aortic cross-clamping, cerebral and somatic NIRS values can be informative, whereas SpO2 values are not available [93]. NIRS values are correlated with continuous venous O_2_ saturation (S_cv_O_2_) in the superior vena cava without, however, replacing it [94]. Chakravarti et al. [95] showed that in post-cardiac surgery patients a cerebral and/or renal NIRS value of <65 % was predictive of a lactate level of over 3 mmol/L which in turn reflects tissue hypoperfusion related to reduced cardiac output. Other authors have shown that in neonates and infants with congenital heart disease requiring surgery or catheterization, the splanchnic NIRS value was correlated with SvO2, lactate levels, and gastric pH [96]. Recent case reports have demonstrated the usefulness of cerebral and renal NIRS as a guide for resuscitation of children in cardiac arrest [97, 98].

### Usefulness of invasive hemodynamic monitoring

#### Usefulness of arterial oxygen pressure (P_a_O_2_)/central venous pressure (CVP) monitoring in cardiogenic shock

- It is advised to monitor S_a_O2 and P_a_O_2_ values in patients with severe respiratory diseases, some of which can be associated with or cause severe heart failure (*strong agreement*).
- It is advised to monitor P_a_O_2_ by measuring arterial blood gas in patients with decompensated circulatory failure (*strong agreement*).
- Vascular access should be obtained in patient with cardiogenic shock by inserting a central venous catheter, preferably in the superior vena cava (*strong agreement*).
- CVP values (and moreover CVP kinetics in a given patient) can provide information on the preload reserve in children with cardiogenic shock (*strong agreement*).

If possible CVP measurements should be performed at the end of expiration, and without mechanical ventilation running (apnea). According to some authors, if the same values are obtained with and without mechanical ventilation, subsequent measurements can be performed with ventilation [70, 99]. Usual CVP values range from 2 to 8 cm H_2_O (1–6 mm Hg). Return to normal CVP values is one of the goals of sepsis management in children [67]. The CVP value provides information on right ventricular preload; however, the relevance of this measurement in unstable, intubated, and ventilated children is limited.

#### Usefulness of central venous oxygen saturation (S_cv_O_2_) monitoring in cardiogenic shock

- We strongly recommend that S_cv_O_2_ be measured continuously or discontinuously in the superior vena cava during cardiogenic shock (*strong agreement*).
- We strongly recommend aiming to reach an upper target value of 70 % (which reflects normal arterial oxygen transport). A value <65–70 % with persistent clinical and laboratory signs of shock can reflect inadequate arterial oxygen transport and should spur efforts to improve oxygen transport by increasing cardiac output and/or transfusion of packed red blood cells (*strong agreement*).
- It is advised to monitor S_a_O2 and P_a_O_2_ values in patients with severe respiratory diseases, some of which can be associated with or can cause severe heart failure (*strong agreement*).

Continuous central venous oxygen saturation (S_cv_O_2_), a variable used to assess microcirculatory and macrocirculatory hemodynamics, provides information on the balance between demand and supply of O_2_ to the tissues. It is measured in the superior vena cava [100] and is one of the goals set by the Surviving Sepsis Campaign (>70 %) [67]. The reliability of S_vc_O_2_ measurements compared with S_V_O_2_ values obtained using pulmonary artery catheters is a subject of discussion. Grissom et al. [101] showed that if the S_cv_O_2_ value measured in the superior vena cava ≥70 % in adults with acute lung injury, then it was unlikely that the mixed venous oxygen saturation (S_v_O_2_) value measured by pulmonary artery catheter would be <60 %. On the other hand, a S_cv_O_2_ value <70 % did not reliably predict a S_V_O_2_ value <60 % (positive predictive value of 31 %). S_cv_O_2_ measurements overestimate the S_V_O_2_ by approximately 3–8 %. The kinetics of these 2 variables are fairly well, although not perfectly, correlated [102]. Recently, De Oliveira et al. [22] demonstrated that a significant decrease in mortality could be achieved if a target value of S_cv_O_2_ >70 % was implemented in sepsis management in children. S_cv_O_2_ can be considered as an interesting marker while not perfect of the balance between oxygen transport and tissue oxygen consumption. In case of shock, and particularly in case of septic shock, changes and heterogeneity of regional and tissue flows should give cautious to an analysis of S_cv_O_2_ value alone.

#### Usefulness of invasive arterial pressure monitoring in cardiogenic shock

- We strongly advise use of an arterial catheter to measure arterial blood pressure in patients with decompensated circulatory failure as seen in cardiogenic shock (*strong agreement*).

Radial access (palmar arch) should be preferred in children even if the validity of the Allen test is not unequivocal. Arterial catheters allow unstable children to be monitored, especially if receiving inotropes or vasopressors [70]. Intraoperative and postoperative monitoring during cardiac surgery or other prolonged or potentially hemorrhagic surgical procedures requires hemodynamic measurements with invasive arterial pressure readings.

#### Usefulness of pulmonary arterial pressure/SvO_2_ monitoring in cardiogenic shock

- In patients with cardiogenic shock who do not respond to first-line treatment, pulmonary artery catheter placement, if necessary, should be performed by a team familiar with this technique (*strong agreement*).
- Routine pulmonary artery catheter placement is not recommended in children with cardiogenic shock (*strong agreement*).

Pulmonary artery catheter is only rarely used in children even if their placement in adults is the standard technique for measuring cardiac output [103]. Pulmonary artery catheter measures the heart chamber pressures and cardiac output using thermodilution. Pulmonary artery catheter also provides continuous information about the mixed venous oxygen saturation (S_v_O_2_) and right ventricular ejection fraction and enables placement of an electrosystolic probe in the right ventricle. Finally, pulmonary artery catheter can be used to perfuse the patient and collect blood samples from each heart chamber. Additional file 1: Table S1 summarizes the normal values of variables measured or calculated using a pulmonary artery catheter [104]. The variation in the values measured using the pulmonary artery catheter provides information on the patient’s hemodynamic and circulatory status. Right atrium pressure increases with hypervolemia, right ventricular dysfunction, or increased juxtacardiac pressure (pericarditis, pneumothorax, and elevated positive expiratory pressure). Pulmonary artery pressure increases with pulmonary arterial hypertension, left ventricular dysfunction, pulmonary embolization, intracardiac shunts (heart disease or lung disease), and positive expiratory pressure and, in contrast, decreases with hypovolemia, shock, right ventricular impairment, and lung failure. Pulmonary arterial wedge pressure increases with hypervolemia, left ventricular impairment, increased left ventricular afterload, and increased juxtacardiac pressure and decreases with hypovolemic, distributive, and septic shock.

#### Usefulness of thermodilution cardiac output monitoring in cardiogenic shock

- Cardiac output determination by thermodilution/pulse contour analysis using a PICCO^®^-type system may be considered in patients with cardiogenic shock refractory to first-line treatment (*strong agreement*).

Cardiac output determination by thermodilution has been developed in adults using a pulmonary artery catheter (Pulmonary Arterial ThermoDilution). Less invasive methods have also been developed to determine the cardiac output by thermodilution using a central venous catheter and an arterial catheter (TransPulmonary ThermoDilution, TPTD). Such methods have been validated in animals [105] and children [106, 107], in particular by comparison with Pulmonary Arterial ThermoDilution values or using the Fick equation. Two methods exist: the PICCO™ system (Pulsion Medical Systems, Munich, Germany) which uses thermodilution + waveform and the LiDCO™ system (LiDCO Ltd, Cambridge, UK) which uses dilution + PulseCO. The PICCO™ system uses a solution of cold saline or dextrose (thermodilution) at 13 °C less than the body temperature (in practice often close to 0 °C) that is injected via the central catheter (subclavian or internal jugular) and detected by a thermistor placed in an arterial catheter in the femoral area. This method is easy to set up but requires frequent calibration. The LiDCO™ system uses a solution of lithium chloride (lithium dilution) which has the advantage that it can be injected via a peripheral venous catheter. The solution is then detected by a sensor on a catheter placed in the radial artery [108]. Cardiac output determinations using the LiDCO™ system are very well correlated with Pulmonary Arterial ThermoDilution measurements [108] as well as with values obtained using the PICCO™ system [103]. However, the use of LIDCO has a very limited experience in childhood and has not been validated yet in such circumstances.

## Field of application 4: circulatory support (indications, techniques, organization, and transfer criteria)

First-line circulatory support in children with cardiogenic shock is venoarterial extracorporeal membrane oxygenation (ECMO). In 1992, del Nido et al. [109] suggested using ECMO for cardiac arrest (extracorporeal cardiopulmonary resuscitation) at an early stage during the low cardiac output phase to reduce neurological complications. ECMO maintains tissue oxygenation pending recovery of heart function. Frequent use of ECMO following cardiac surgery for congenital or acquired cardiomyopathy in pediatric patients has led care facilities to purchase the specialized equipment needed for this technique.

### Usefulness of arteriovenous extracorporeal membrane oxygenation for the management of cardiogenic shock in children

- We strongly recommend using ECMO in patients with cardiogenic shock refractory to conventional therapy (*strong agreement*).

No prospective randomized studies have been carried out on pediatric populations to compare morbidity and mortality of patients with cardiogenic shock or cardiac arrest with or without circulatory support by ECMO. Analysis of the Extracorporeal Life Support Organization (ELSO) registry (https://www.elso.org/), which in July 2013 included information on 55,668 patients, shows that the use of ECMO has decreased since 1992 in neonates and pediatric patients for respiratory indications (since when inhaled nitric oxide has been used in clinical practice), but has increased continuously over the same period for cardiac indications. Two main indications are observed: severe heart failure or cardiogenic shock and cardiac arrest. Delayed use of ECMO in patients with cardiogenic shock increases the risk of cardiac arrest. Numerous retrospective studies [110–120], as well as data from the ELSO registry (https://www.elso.org/), show that when ECMO is used the mean patient survival is >40 % for cardiogenic shock and >35 % for cardiac arrest. The prognosis for myocarditis is very good if ECMO is started prior to cardiac arrest [121–124]. The complications associated with ECMO are mostly neurological: electrical and clinical seizure, cerebral infarction and bleeding, brain death. Depending on patient populations, Brown et al. [121] reported persisting low-to-moderate disabilities in 12–50 % of patients treated with ECMO. In 16 survivors of a series of 39 children treated with ECMO, Lequier et al. [125] reported a mean score of 73 ± 16 on the Bayley scale at 2 years of age. Eight patients showed moderate mental retardation.

### Extracorporeal membrane oxygenation in patients with cardiac arrest

- We strongly recommend using ECMO in patients with cardiac arrest within ≥15 min and <60 min (*strong agreement*).

According to International Liaison Committee on Resuscitation, use of ECMO should be considered in children with cardiac arrest refractory to conventional cardiopulmonary resuscitation, if cardiac arrest occurred in a highly monitored setting and the necessary support equipment and expertise are available to implement the technique rapidly [126]. In a series of 682 patients with a median age of 3 months (ELSO registry) treated with extracorporeal cardiopulmonary resuscitation [127], the survival rate after discharge was 38 %. This study suggests that a pH < 6.9 prior to ECMO is significantly associated with death within 72 h or a poor neurological prognosis. The meta-analysis performed by Tajik and Cardarelli [128] of 37 studies (288 patients) including case reports and observational pediatric studies on patients with cardiac arrest treated with extracorporeal cardiopulmonary resuscitation, showed that survival rate was very variable: It ranged from 0 to 100 % in small centers that had low numbers of patients and from 6 to 79 % for the larger cohorts. Matos et al. [116] demonstrated that morbidity and mortality increase if cardiopulmonary resuscitation lasts >15 min and that conventional resuscitation reaches its limits with a likelihood of survival of 41 % for cardiopulmonary resuscitation lasting 1–15 min, but only 12 % if cardiopulmonary resuscitation lasts >35 min. The neurological prognosis of survivors is good and reaches 70 % when cardiopulmonary resuscitation lasts <15 min and 60 % if CPR lasts <35 min. The survival of children undergoing cardiac surgery is 38.5 % if cardiopulmonary resuscitation lasts >35 min and ECMO is used, but drops to 16.7 % if ECMO is not used (*p* < 0.0001). Turek et al. [120] reported that if ECMO was implemented within 40 min using a previously prepared circuit, neurological complications were decreased by 52 % (*p* < 0.04) compared with the conventional method (>40 min). However, no decrease in the mortality rate was observed. In a series of 42 children treated with extracorporeal cardiopulmonary resuscitation, Delmo-Walter et al. [112] showed a difference of mortality depending on the duration of cardiopulmonary resuscitation prior to implementation of ECMO: 30 ± 1.3 min vs. 46 ± 4.2 min (*p* = 0.003). The faster the limits of cardiopulmonary resuscitation are recognized, the earlier ECMO should be initiated, thereby limiting mortality and organ lesions [129].

### Usefulness of mechanical ventricular assist devices in the management of cardiogenic shock in children

- We recommend that the use of ventricular assist devices in pediatric patients with cardiogenic shock refractory to conventional therapy be discussed with cardiologists (*strong agreement*).

No prospective randomized studies have been performed to compare the morbidity and mortality of children with cardiogenic shock treated with or without a ventricular assist device. In a single-center retrospective study including 16 children with fulminant myocarditis, Wilmot et al. [124] reported a survival rate of 75 % at discharge whatever the support method used (ventricular assist device or ECMO).

### Usefulness of extracorporeal membrane oxygenation implementation criteria for children with cardiogenic shock

- To lower the risks of mortality and neurological morbidity, we strongly recommend implementing ECMO when pH ≥ 7.2 and lactate < 9 mmol/L and using low-to-moderate inotrope support (*strong agreement*).
- The decision to implement ECMO in children with cardiogenic shock is based on clinical, laboratory, and prognostic criteria, the relevance of which must be considered jointly by pediatric cardiologists and intensive care physicians (*strong agreement*).

No prospective randomized studies have been performed to define specific criteria for ECMO implementation in children with cardiogenic shock. Based on data from the ELSO registry in 2005, Thiagarajan et al. [127] reported a mean pre-ECMO arterial pH of 7.26 (7.06–7.38) for survivors and 7.17 (6.9–7.36) for non-survivors (*p* = 0.001). Multivariate analysis demonstrated that the mean pre-ECMO pH was <6.9 in the group of children with the highest mortality. For the same patient population, Barrett et al. [130] demonstrated that neurological prognosis was poorest for children with a pH < 6.865. Two studies by Huang et al. [114, 131], which included 27 and 54 cases of extracorporeal cardiopulmonary resuscitation and 41 and 46 % of survivors, respectively, revealed that pre-ECMO lactate levels were statistically higher in non-survivors than in survivors [for the first study 14 (10.2–19.6) vs. 8.5 (4.4–12.6) mmol/L, *p* < 0.01 and for the second study 13.4 ± 6.4 vs. 8.8 ± 5.1 mmol/L, *p* < 0.01]. In 218 post-cardiac surgery pediatric patients, Trittenwein et al. [132] reported that the predicted postoperative mortality was 80 % if the patient showed arterial lactate levels >7.7 mmol/L with S_cv_O_2_ <60 %, or if arterial lactate levels >18 mmol/L and S_cv_O_2_ >60 % on admission to intensive care (*p* < 0.05). The authors suggest that these levels be used as postoperative criteria for ECMO implementation.

### Usefulness of mobile circulatory support units for implementing extracorporeal membrane oxygenation in patients with cardiogenic shock far from an expert center

- ECMO should be implemented by a team of trained healthcare professionals (*strong agreement*).
- Patients treated with ECMO should be transferred to an expert center (*strong agreement*).

No randomized studies have been performed on the use of mobile circulatory support units. Before requesting a mobile circulatory support unit, the child’s status (cardiogenic shock/cardiac arrest), on-site therapeutic means and hemodynamic assessment measures, and the response time of the mobile circulatory support unit should be taken into account.

### Usefulness of expert centers for managing pediatric patients with cardiogenic shock treated with venoarterial extracorporeal membrane oxygenation

- Candidate pediatric ECMO reference centers should perform at least 15–20 ECMOs on pediatric patients per year (*strong agreement*).

No randomized studies have been performed on expert pediatric ECMO centers.

An expert center must have a multidisciplinary technical platform that can ensure complete care of pediatric patients treated with ECMO for both medical and surgical indications. In a study based on 3867 pediatric congenital cardiopathy patients treated with ECMO, Karamlou et al. [133] showed that low in-hospital mortality was associated with centers that managed large numbers of ECMO cases (>30/year; *p* = 0.01), whereas low ECMO activity (<15/year) was a risk factor for in-hospital mortality (OR 1.75; CI 95 % 1.03–2.94; *p* = 0.03).

## Conclusion

**Fig. 2:**
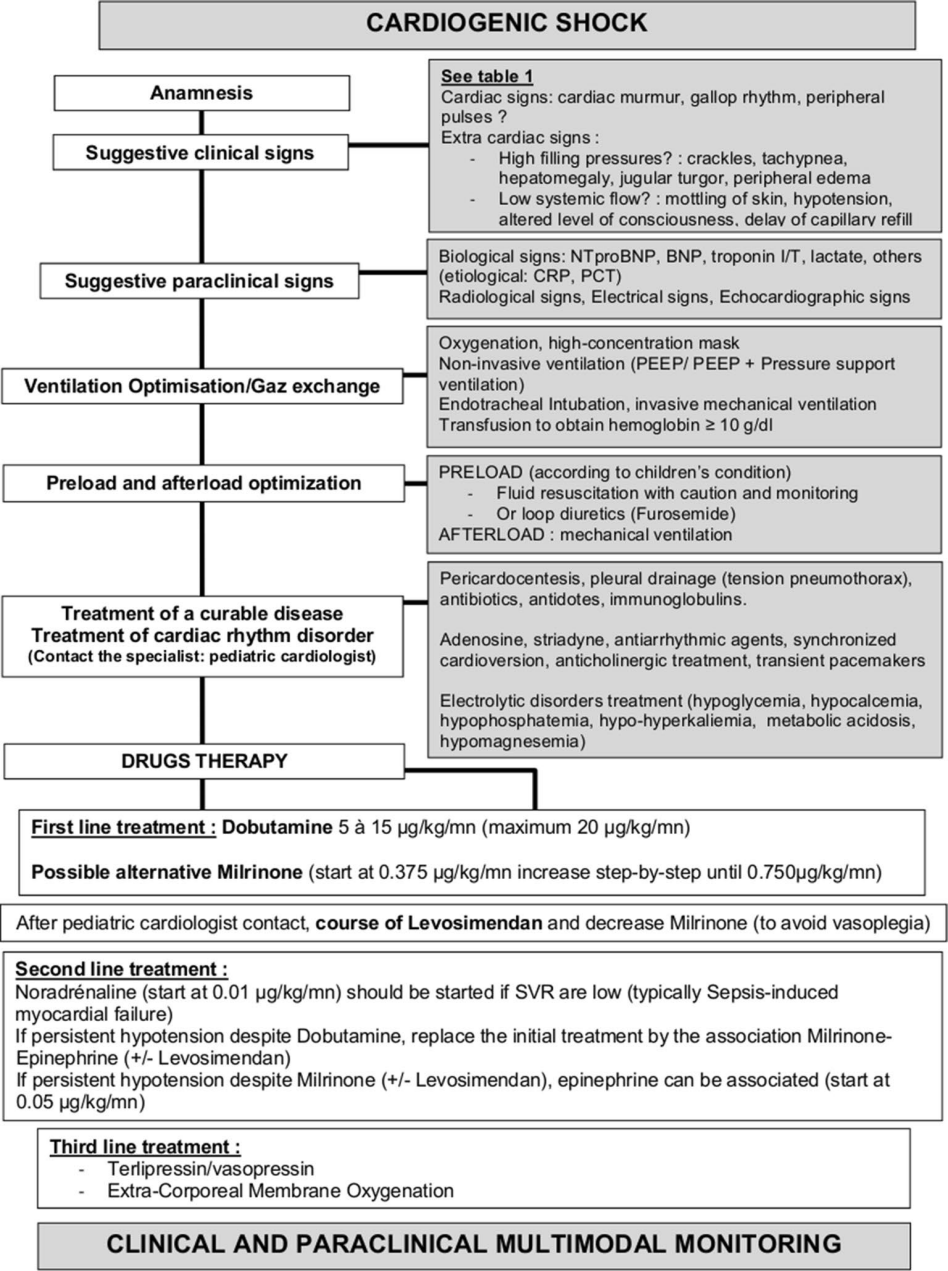
Decision tree for the management of children with a cardiogenic shock. (*PEEP* positive end expiratory pressure, *SVR* systemic vascular resistance)

We report here experts’ recommendations regarding management of children with cardiogenic shock. We hope this work will help healthcare professionals in their daily practice. We propose in conclusion a decision tree (Fig. 2) for the management of cardiogenic shock in children by referring to recommendations we have published.

## Additional file

10.1186/s13613-016-0111-2 Hemodynamic parameters provided by Pulmonary Artery Catheter (RA : right atrium; RAP: right atrium pressure; RV : right ventricle, LV : left ventricle, SVR : systemic vascular resistance, PVR : pulmonary vascular resistance, CO : cardiac output, SvO_2_ : venous oxygen saturation, SaO_2_ : arterial oxygen saturation, VO_2_ = O_2_ consumption, HR : heart rate, BSA : body surface area, M : mean, Hb : oxygen tension in venous blood, PaO_2_ : oxygen tension in arterial blood, rSO_2_ : regional tissue oxygenation).
