# Supplementary material for: Experts’ recommendations for the management of cardiogenic shock in children
Source: Ann Intensive Care. 2016 Feb 16;6:14. doi: 10.1186/s13613-016-0111-2 (PMC4754230; doi:10.1186/s13613-016-0111-2)
Supplement: Supplementary file 1 — 10.1186/s13613-016-0111-2 Hemodynamic parameters provided by Pulmonary Artery Catheter (RA : right atrium; RAP: right atrium pressure; RV : right ventricle, LV : left ventricle, SVR : systemic vascular resistance, PVR : pulmonary vascular resistance, CO : cardiac output, SvO2 : venous oxygen saturation, SaO2 : arterial oxygen saturation, VO2 = O2 consumption, HR : heart rate, BSA : body surface area, M : mean, Hb : oxygen tension in venous blood, PaO2 : oxygen tension in arterial blood, rSO2 : regional tissue oxygenation). [file 13613_2016_111_MOESM1_ESM.doc]

Table S1: Hemodynamic parameters provided by Pulmonary Artery Catheter (RA : right atrium; RAP: right atrium pressure; RV : right ventricle, LV : left ventricle, SVR : systemic vascular resistance, PVR : pulmonary vascular resistance, CO : cardiac output, SvO_2_ : venous oxygen saturation, SaO_2_ : arterial oxygen saturation, VO_2_ = O_2_ consumption, HR : heart rate, BSA : body surface area, M : mean, Hb : oxygen tension in venous blood, PaO_2_ : oxygen tension in arterial blood, rSO_2_ : regional tissue oxygenation).

| Measures | Normal values |  |
| --- | --- | --- |
| RAP = RV filling pressure if no valvulopathy) | 4 ± 4 mm Hg |
| Pulmonary Arterial Pressure (PAP) | Systolic: 15 to 25 mm Hg ; Diastolic: 8 to 15 mm Hg; Mean: 14 ± 3 mm Hg |
| Pulmonary Artery Occlusion Pressure (PAOP)  or Pulmonary Artery Wedge Pressure (PAWP) | 4 to 12 mm Hg  LV filling pressure |
| CO by thermodilution | 4 – 9 years: 4.6 ±1.1 L/min; > 10 years: 6.8±1.2 L/min | Formula |
| Mixed venous oxygen saturation (S_v_O_2_) | 65 à 75 % | SaO_2_- VO_2_/(COx1.34x[Hb]) |
| Cardiac Index  Stroke Volume Index SVI  Stroke Volume  LV stroke Work Index (LVSWI)  RV stroke Work Index (RVSWI)  SVR Index  PVR Index | 3.3 to 6;3.5 to 5.5; 3.2 ±0.2 L/mn/m² according to authors  30 to 60 mL/m²  50 to 80 mL/beat  35 to 47 g.m/m²  6 to 9 g.m/m²  2180 ± 210 dyne/sec/cm^-5^/m²  240 ± 45 dyne/sec/cm^-5^*/m²* | CO/BSA  CI/HR  CO/HR  SVI x (MAP – PAOP) x 0.0136  SVI x (MPAP – RAP) x 0.0136  (MAP – RAP)/BSA x 79.9  (MPAP – PAOP)/BSA x 79.9 |
| Oxygenation parameters  - Venous Oxygen Content: CvO_2_  - Arterial Oxygen Content: CaO_2_  - Oxygen Consumption Index: VO_2_I  - Oxygen Extraction Ratio: EO_2_ | 10 to 15 mL of O_2_ /dL  17 to 20 mL of O_2_ /dL  100 to 130 - 160 to 180 mL/mn/m² (child - neonate)  24 to 28% | (1.34 x Hb x SvO_2_ )+ (PvO_2_ x 0.003)  (1.34 x Hb x SaO_2_ )+ (PaO_2_ x 0.003)  CI x (CaO_2_ – CvO_2_)  (CaO_2_ – CvO_2_)/CaO_2_ |
| NIRS  Cerebral rSO2  Somatic rSO2 | 60±10%  75±10% |  |
